# Machine learning-based survival rate prediction of Korean hepatocellular carcinoma patients using multi-center data

**DOI:** 10.1186/s12876-022-02182-4

**Published:** 2022-02-27

**Authors:** Byeonggwan Noh, Young Mok Park, Yujin Kwon, Chang In Choi, Byung Kwan Choi, Kwang il Seo, Yo-Han Park, Kwangho Yang, Sunju Lee, Taeyoung Ha, YunKyong Hyon, Myunghee Yoon

**Affiliations:** 1 Division of Hepatobiliary and Pancreas Surgery, Department of Surgery, Biomedical Research Institute, Pusan National University College of Medicine, 179 Gudeok-Ro, Seo-Gu, Busan, 49241 Republic of Korea; 2 Department of Surgery, Seoul Medical Center, Seoul, Republic of Korea; 3 Division of Gastroenterology, Department of Surgery, Pusan National University College of Medicine, Busan, Republic of Korea; 4 Department of Neurosurgery, Pusan National University College of Medicine, Busan, Republic of Korea; 5 Division of Hepatology, Department of Internal Medicine, Kosin University College of Medicine, Busan, Republic of Korea; 6 Department of Surgery, College of Medicine, Inje University, Busan Paik Hospital, Busan, Republic of Korea; 7 Division of Hepatobiliary Pancreas and Transplant Surgery, Department of Surgery, Pusan National University, Yangsan Hospital, Republic of Korea; 8 Division of Medical Mathematics, National Institutes for Mathematical Sciences, Daejeon, Republic of Korea

**Keywords:** Machine learning, Hepatocellular carcinoma, Survival time, Multi-center data

## Abstract

**Aim:**

To predict survival time of Korean hepatocellular carcinoma (HCC) patients using multi-center data as a foundation for the development of a predictive artificial intelligence model according to treatment methods based on machine learning.

**Methods:**

Data of patients who underwent treatment for HCC from 2008 to 2015 was provided by Korean Liver Cancer Study Group and Korea Central Cancer Registry. A total of 10,742 patients with HCC were divided into two groups, with Group I (2920 patients) confirmed on biopsy and Group II (5562 patients) diagnosed as HCC according to HCC diagnostic criteria as outlined in Korean Liver Cancer Association guidelines. The data were modeled according to features of patient clinical characteristics. Features effective in predicting survival rate were analyzed retrospectively. Various machine learning methods were used.

**Results:**

Target was overall survival time, which divided into approximately 60 months (= /< 60 m, > 60 m). Target distribution in Group I (total 514 samples) was 28.8%: (148 samples) less than 60 months, 71.2% (366 samples) greater than 60 months, and in Group II (total 757 samples) was 66.6% (504 samples) less than 60 months, 33.4% (253 samples) greater than 60 months. Using NG Boost method, its accuracy was 83%, precision 84%, sensitivity 95%, and F1 score 89% for more than 60 months survival time in Group I with surgical resection. Moreover, its accuracy was 79%, precision 82%, sensitivity 87%, and F1 score 84% for less than 60 months survival time in Group II with TACE. The feature importance with gain criterion indicated that pathology, portal vein invasion, surgery, metastasis, and needle biopsy features could be explained as important factors for prediction in case of biopsy (Group I).

**Conclusion:**

By developing a predictive model using machine learning algorithms to predict prognosis of HCC patients, it is possible to project optimized treatment by case according to liver function and tumor status.

## Introduction

Hepatocellular carcinoma (HCC) is characterized as a disease that spreads throughout the liver due to repeated intrahepatic recurrence of localized lesions, resulting in death due to liver failure. HCC typically originates from underlying liver disease and the major cause is hepatitis B or C virus infection with or without cirrhosis [1].

Alcohol abuse and cigarette smoking are also common factors of etiology, while metabolic diseases including obesity and diabetes as well as nonalcoholic fatty liver disease become amplifiers of risk of HCC [2]. There are various treatment methods for HCC. and it is necessary to predict the survival period and survival rate following treatment methods. Hepatic resection is the best treatment option for potential curative outcomes, but less than one-third of HCC cases are eligible for resection of HCC at the time of diagnosis [3]. In addition, the high rate of recurrence despite curative resection presents a major challenge in HCC management [4]. Most postoperative recurrence cases occur in the remnant liver as intrahepatic recurrence [5], and discerning reliable predictors is essential for patient risk evaluation, treatment decision-support and long-term survival improvement. HCC can be diagnosed with biopsy or with noninvasive imaging in high risk groups with chronic hepatitis or cirrhosis. If the imaging diagnosis is indecisive or has atypical features, biopsy is suggested. However, in case of patients with ascites, high risk of bleeding, and HCC in challenging location, biopsy is difficult, and therefore imaging diagnosis is preferred in these cases [6]. For the reason, it is necessary to predict the survival period and survival rate following treatment methods. HCC can be diagnosed on biopsy or by noninvasive imaging in high risk groups with chronic hepatitis or cirrhosis. If the imaging diagnosis is indecisive or has atypical features, biopsy is recommended. However, biopsy is difficult in patients with ascites, high risk of bleeding, or HCC in a challenging location, and in such cases, imaging diagnosis is preferred [6]. For the reason, it is necessary to predict the survival period and survival rate following treatment methods.

In order to develop a predictive model for the survival period and survival rate, we might need to obtain multi-center data, which is a sufficient number to represent the population, and including well curated features for analyzing HCC and survival period. To do this with overcoming internal data limitations in hospital, we utilized the HCC multi-center data of Korea Central Cancer Registry, National Cancer Center, and Ministry of Health and Welfare data sets, and appropriate machine learning algorithms. This artificial intelligent type predictive model could lead us to develop personalized treatment methods that consider liver function and HCC status, and data-based treatment imposing clinician's insights.

Various machine learning algorithms were used for survival rate prediction, which are voting ensembles, Logistic Regression, K-nearest neighbors, Decision Tree Classifier, Support Vector Machine, Random Forest, Extreme gradient boosting trees (XG Boost), Light GBM, and Natural Gradient Boosting (NG Boost).

The aim of this study was machine learning-based survival rate prediction of Korean hepatocellular carcinoma patients using the multi-center data as a foundation for development of a new predictive artificial intelligence model according to treatment methods.

## Methods

### Patients

**Fig. 1:**
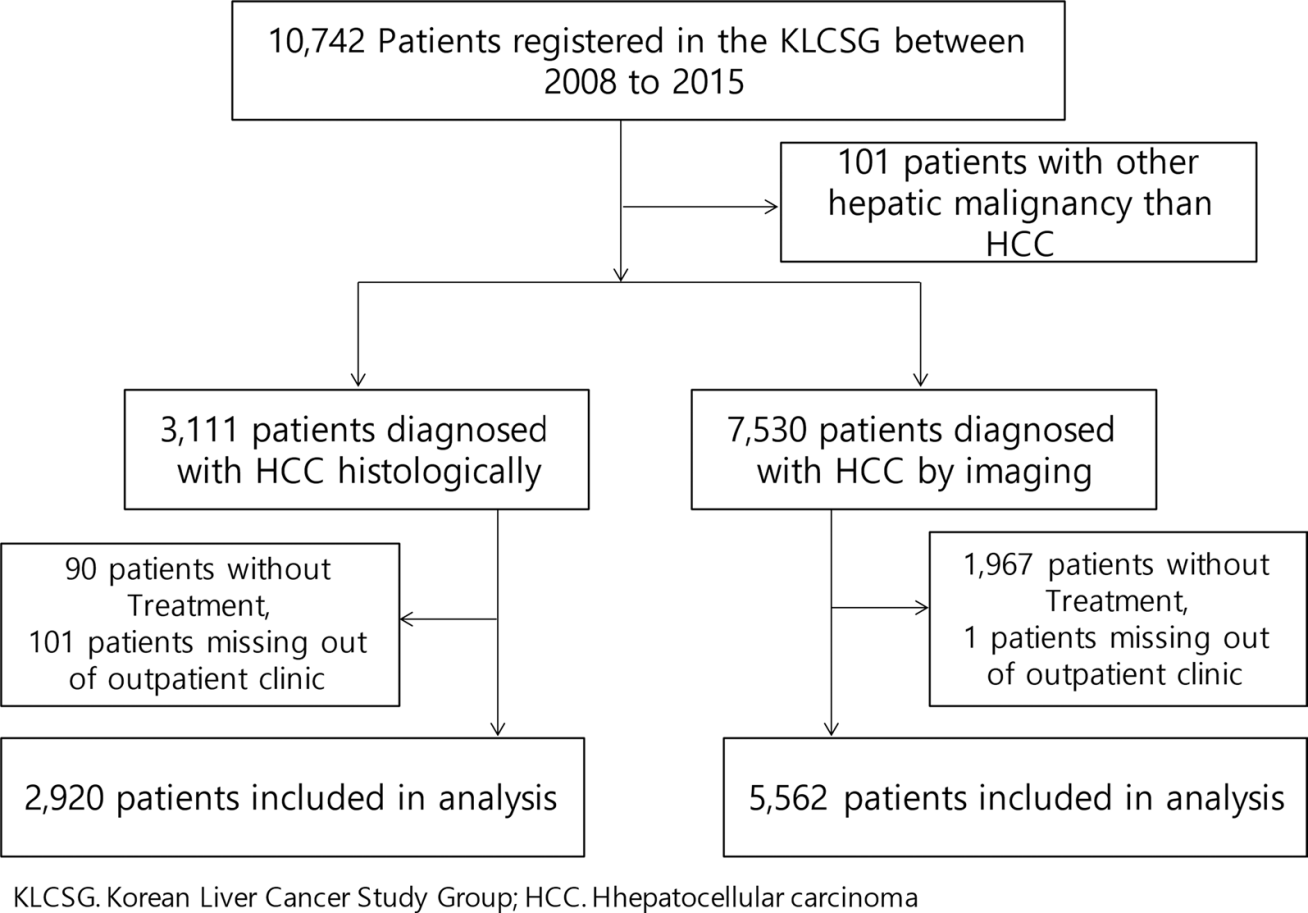
Flow diagram of the patients population

A total of 10,742 patients diagnosed with liver cancer, as registered by Korean Liver Cancer Study Group and Ministry of Health & Welfare, Korea Central Cancer Registry from 2008 to 2015, were evaluated; 101 patients had diagnoses of liver cancer other than HCC and were excluded (Fig. 1). Cases were divided into Group I diagnosed as HCC before treatment, and Group II diagnosed according to HCC diagnostic criteria as outlined in Korean Liver Cancer Association guidelines [6]. HCC is diagnosed if the histological and immunological findings after biopsy are positive or if the image findings are consistent with HCC, at a size larger than or equal to 1 cm, hyper enhancement in arterial phase and washout at portal venous or delayed phase on multi-phase CT and MRI using specific contrast, in high-risk patients. The authors divided the patients according to diagnostic modality, 2,920 patients were analyzed with HCC histologically either by needle or surgical biopsy (Group I) and 5,562 patients were included with HCC radiologically (Group II) (Fig. 1), with baseline demographic data previously published [7].

The study design was approved by the Institutional Review Board of Pusan National University Hospital (No. 2009-025-095) and was conducted in accordance with the Declaration of Helsinki.

### Feature selection

Using predictive algorithms based on machine learning, the data on HCC patients collected at Korea Central Cancer Registry were used to determine the appropriate treatment (Table 1) and survival period for HCC patients with a range in liver functionality. The authors attempted to determine features that are effective in predicting survival rate and to interpret said features in keeping with the purpose of this study.

**Table 1:** Treatment Methods. Group I, patients who were diagnosed histologically; Group II, patients who were diagnosed radiologically. a: radiofrequency ablation, alcohol injection, other local ablation, b: transarterial chemoembolization with gelfoam, beads, chemolipiodolization, chemoinfusion, radioembolization

Treatment methods	Group I (n = 2920)	Group II (n = 5562)
Surgical resection (n, %)	1,889(64.7)	20(0.4)
Liver TPL (n, %)	102(3.5)	3(0.1)
Local ablation ^a^ (n, %)	184(6.3)	989(17.8)
Transarterial therapy ^b^ (n, %)	593(20.3)	3,994(71.8)
Chemotherapy (n, %)	112(3.8)	422(7.6)
Radiation therapy (n, %)	35(1.2)	110(2.0)
No treatment	5(0.2)	24(0.4)

First, the process of pre-processing the data was conducted as previously explained. The analysis index of the collected data was a total of 117 features including image features and BCLC stage (Table 2). Also, Height and weight, liver function test, liver cirrhosis status, radiologic TNM findings, and histopathological TNM findings (Table 3) were included. Therefore, we used the 51 features in the biopsy (Group I) and 62 features when biopsy is not performed (Group II). In prediction of survival time rate according to treatment methods, 57 features, 48 features were used in TACE and surgical resection, respectively. After the exception is also feature more than the absolute value of the Correlation of 0.9 it was finally classified according to the treatment method the feature used.

**Table 2:** BCLC Staging. Group I, patients who were diagnosed histologically; Group II, patients who were diagnosed radiologically

BCLC staging	Group I (n = 2920)	Group II (n = 5562)
Stage 0 (n, %)	169(5.8)	445(8.0)
Stage A (n, %)	1,455(49.8)	1,966(35.3)
Stage B (n, %)	277(9.5)	613(11.0)
Stage C (n, %)	674(23.1)	1,737(31.2)
Stage D (n, %)	47(1.6)	194(3.5)
Undefined (n, %)	298(10.2)	607(10.9)

**Table 3:** TNM Staging. Group I, patients who were diagnosed histologically; Group II, patients who were diagnosed radiologically

TNM stage*	Group I (n = 2920)	Group II (n = 5562)
Stage I (n, %)	329(11.3)	1,065(19.1)
Stage II (n, %)	1,512(51.8)	1,875(33.7)
Stage III (n, %)	438(15.0)	1,507(27.1)
Stage IV-A (n, %)	60(2.1)	631(11.3)
Stage IV-B (n, %)	50(1.7)	456(8.2)
Undefined (n, %)	531(18.2)	28(0.5)

**Table 4:** Target point and data processing of machine learning

Target	Explanation
Mortality	0: Alive 1: Expired
Overall survival time	Death or Last follow up date—First treatment date
Treatment	1: Surgical resection 2: Liver transplantation 3: Local ablation therapy(RFA) 4: Transarterial therapy (TACE) 5: Others 9,999,999: missing data

**Table 5:** Train set and test set of group I

	Counts (train)	Counts (test)	Ratio (train)	Ratio (test)
0	1,122	267	45.205479	42.995169
1	1,360	354	54.794521	57.004831

### Data splits for machine learning processing

**Fig. 2:**
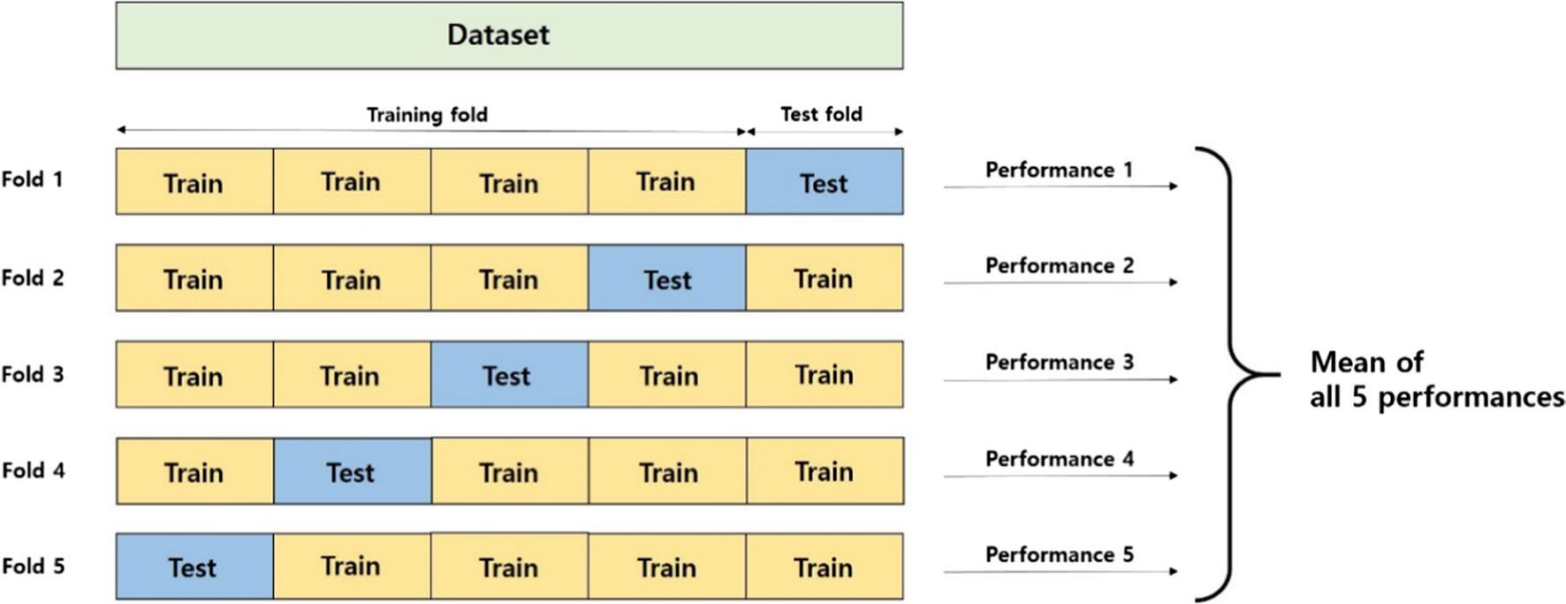
Schematic picture of 5 different prediction procedure same as 5 folds

Because of slightly imbalanced given data, we used stratified sampling with the ratio 8:2 for train and test two disjoint sets, respectively. We performed 5 different predictions (Fig. 2), which is same as fivefold but for test.

And then the average of accuracy, precision, sensitivity and F1 score were obtained from the 5 predictions.

### Machine learning method

In this study, various machine learning algorithms were used for survival rate prediction according to mortality, survival time, and treatment method. The algorithms are voting ensembles [8–11], Logistic Regression (LR) [12], K-nearest neighbors (KNN) [13, 14], Decision Tree (DT) Classifier [15–17], Support Vector Machine (SVM) [18–21], Random Forest (RF) [22, 23], Extreme gradient boosting trees (XG Boost) [24], Light GBM [25, 26], and Natural Gradient Boosting (NG Boost) [27, 28]. Its prediction results are compared in Tables 6, 8, and 10.

**Table 6:** Train set and test set of group II before down sampling

	Counts (train)	Counts (test)	Ratio (train)	Ratio (test)
0	1099	264	18.353373	17.635271
1	4889	1233	81.646627	82.364729

**Table 7:** Prediction of mortality rate of Group I according to Machine learning methods

Classifier	Voting	LR	KNN	DT	SVM	RF	XGBoost	LightGBM	NGBoost
Accuracy	0.6828	0.6908	0.6329	0.6763	0.6731	0.6989	0.7279	0.7198	0.7037
Precision	0.6792	0.6878	0.6431	0.6719	0.6658	0.6948	0.7237	0.7169	0.7017
Recall score	0.6822	0.6911	0.6439	0.6742	0.6599	0.6977	0.7268	0.7206	0.7056
F1 score	0.6796	0.6880	0.6328	0.6724	0.6613	0.6954	0.7246	0.7172	0.7015
ROC-AUC	0.6822	0.6911	0.6439	0.6742	0.6599	0.6977	0.7268	0.7206	0.7056

**Table 8:** Prediction of mortality rate of Group II before down sampling according to Machine learning methods

Classifier	Voting	LR	KNN	DT	SVM	RF	XGBoost	LightGBM	NGBoost
Accuracy	0.8216	0.8230	0.7956	0.8236	0.8230	0.8383	0.8363	0.8337	0.8343
Precision	0.5549	0.4118	0.6077	0.6851	0.4118	0.7222	0.7147	0.7076	0.7152
Recall score	0.5018	0.4996	0.5708	0.6399	0.4996	0.6280	0.6372	0.6505	0.5958
F1 score	0.4584	0.4514	0.5796	0.6560	0.4514	0.6523	0.6601	0.6702	0.6158
ROC-AUC	0.5018	0.4996	0.5708	0.6399	0.4996	0.6280	0.6372	0.6505	0.5958

**Table 9:** Train set and test set of group II after down sampling

	Counts (train)	Counts (test)	Ratio (train)	Ratio (test)
0	1080	283	49.541284	51.831502
1	1100	263	50.458716	48.168498

**Table 10:** Prediction of mortality rate of group II after down sampling according to machine learning methods

Classifier	Voting	LR	KNN	DT	SVM	RF	XGBoost	LightGBM	NGBoost
Accuracy	0.6850	0.7179	0.6429	0.7271	0.7033	0.7692	0.7802	0.7601	0.7784
Precision	0.6936	0.7275	0.6585	0.7318	0.7282	0.7708	0.7813	0.7598	0.7788
Recall score	0.6804	0.7137	0.6361	0.7240	0.6967	0.7674	0.7787	0.7595	0.7772
F1 score	0.6777	0.7121	0.6269	0.7237	0.6903	0.7679	0.7792	0.7596	0.7776
ROC-AUC	0.6804	0.7137	0.6361	0.7240	0.6967	0.7674	0.7787	0.7595	0.7772

## Results

The target was overall survival time, which is divided into about by 60 months (= < 60 m, > 60 m) (Table 4). After preprocessing of the given data, the target distributions for each group, were 148 samples (28.8%), whose the overall survival time is less than 60 months, 366 samples (71.2%), greater than 60 months in Group I, which has total 514 samples, and 504 samples (66.6%), less than 60 months, 253 samples (33.4%), 33.4% (253 samples) greater than 60 months in Group II, total 757 samples.

1. Prediction of mortality rate according to the presence or absence of biopsy
2. In case of biopsy (Group I)

**Fig. 3:**
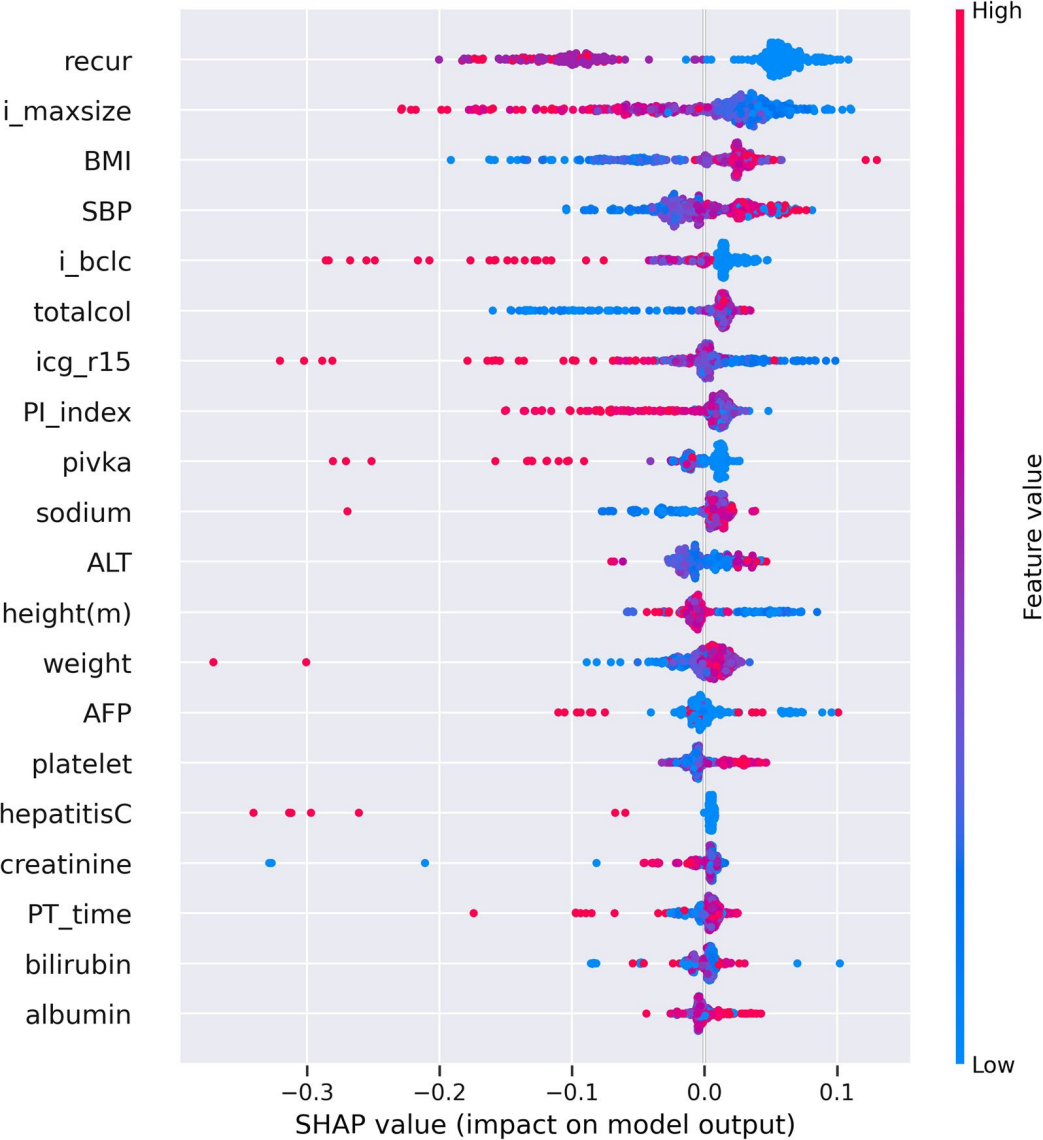
Feature Importance F1 by SHAP values of Group I with surgical resection according to 5 folds shown in Fig. 2, respectively

When biopsy was performed (Group I), it can be seen that the surviving and deceased samples were relatively evenly distributed (Table 5). Therefore, in this case, down sampling or up sampling was not performed. Even in this case, the XG Boost method that obtained the best result among the methods used in the prediction was not significantly lower than the accuracy in precision, recall, and ROC value, but all indicators including accuracy were 70% (Table 6). Among the methods used in prediction, the XG Boost method obtained the best result. Pathology Portal invasion, method surgery, image M, Pathology T, needle biopsy, etc. can be seen as the most important factors for prediction (Fig. 3).

(2) When biopsy is not performed (Group II)

If the biopsy was not performed, the surviving and deceased samples were unevenly distributed (Tables 7, 8), and the down sample was used to obtain the predicted results (Tables 9, 10).

It can be seen that the classification by the XG Boost method has obtained relatively the best results, and it can be seen that the precision, recall, and ROC values ​​are not significantly lower than the accuracy.

Among the methods used in prediction, the XG Boost method obtained the best result, and when looking at the method using GAIN in the importance analysis, image portal invasion, image T, image tumor size, BCLC stage, etc. can be seen as the most important factors for prediction.

Using NG Boost method, its accuracy was 83%, precision 84%, sensitivity 95%, and F1 score 89% for more than 60 months survival time in Group I with surgical resection. Moreover, its accuracy was 79%, precision 82%, sensitivity 87%, and F1 score 84% for less than 60 months survival time in Group II with TACE. The feature importance with gain criterion indicated that Pathology Portal invasion, method surgery, image M, Pathology T, needle biopsy features could be explained as important factors for prediction in case of biopsy (Group I).

2. Prediction of survival time rate according to treatment methods was analyzed.

To analyze the survival rate according to the treatment method, the analysis target was overall survival (Table 4). It was analyzed by dividing the survival period into less and more than 60 months. Five classifications were made among the various treatment methods of the collected data (Class 1: Surgical resection, Class 2: Liver transplantation, Class 3: Local ablation therapy, Class 4: Trans arterial Chemoembolization (TACE), Class 5: Others). Among the treatment methods, the prediction between liver transplantation and local ablation therapy was inaccurate. The problem often lies with too little data, and treatment method being determined by clinical experience. However, in the case of predicting only surgical resection and TACE, a model with good results of high accuracy and precision was developed (Table 4).

**Fig. 4:**
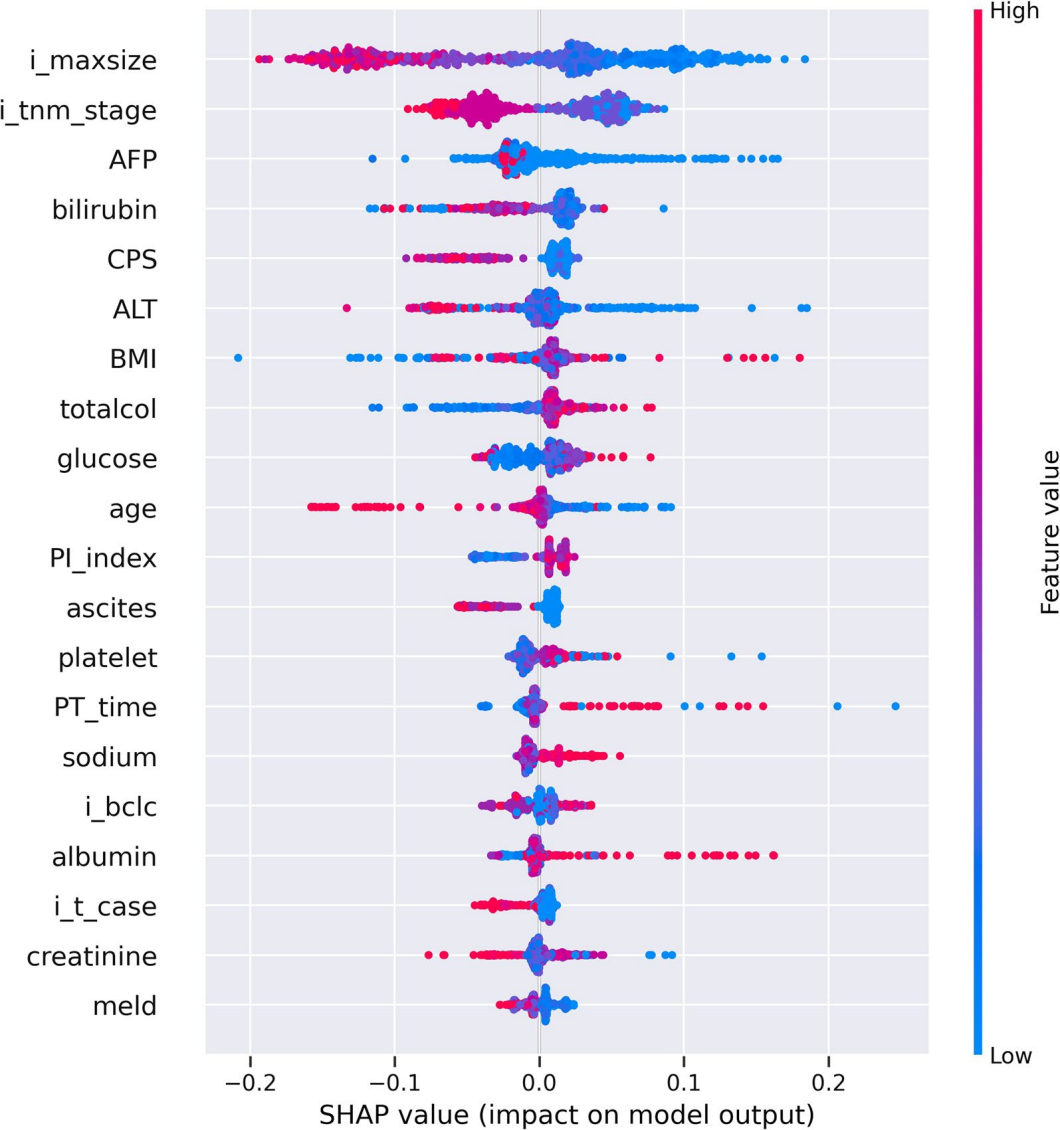
Feature Importance F1 by SHAP values of Group II with TACE according to 5 folds shown in Fig. 2, respectively

Significant treatment methods were TACE and surgical resection. According to these two treatment methods, survival rate analysis (Tables 11, 12) was performed with features (Figs. 3, 4).

**Table 11:** Prediction results for survival rate of group I with surgical resection: the results were obtained by the average of fivefold type testing

Data Set	Accuracy (%)	Precision (%)	Sensitivity (%)	F1 score (%)
< = 60 m	83	82	55	66
> 60 m		84	95	89

**Table 12:** Prediction results for survival rate of Group II with TACE: the results were obtained by the average of fivefold type testing

Data set	Accuracy (%)	Precision (%)	Sensitivity (%)	F1 score (%)
< = 60 m	79	82	87	84
> 60 m		71	62	66

## Discussion

The value of multi-center data will depend on the degree of standardization of the collected data. In addition, it must include a sufficient number to represent the population. Using a machine learning-based prediction algorithm, the authors analyzed the appropriate treatment for HCC and the properties that influence the survival period accordingly. Through this work, the authors intended to develop an algorithm that can propose the optimal personalized treatment for each individual according to liver function and HCC condition. Recently, computer-based diagnosis and prognostic prediction by machine-learning algorithms and deep-learning systems have been widely used and more individualized prediction based on a combination of variables is provided by nomogram models [29].

By developing machine-learning algorithms and deep-learning systems to predict prognosis of HCC patients, it is possible to offer individualized recurrence surveillance and adjuvant therapy. Data collection in a standardized form is the priority of national big data management. In order to solve the limitations of national multi-center data, collection in a nationally standardized format and uniformity of the processing method of the missing data is essential.

In the results of this study, among the various treatment methods, the prediction of survival rate with liver transplantation and local ablation therapy was inaccurate. The problem often lies with too little data, and treatment method being determined by clinical experience, thus different in each case.

In addition to tumor extent, hepatic reservoir plays a major role when selecting the treatment method. Before the treatment selection, laboratory tests and imaging were performed to evaluate liver function and tumor extent, and great effort was made to combine these factors in order to choose the most suitable treatment modality and predict the prognosis [30].

Generally, late recurrence (more than 2 years) after liver resection for HCC is regarded as a multi-centric tumor or a de novo cancer. Therefore, surveillance for recurrence 2 years after surgery should be targeted to the liver [31]. In this study, machine learning (ML) model was used to evaluate the relationship between preoperative and treatment modalities with treatment results expressed by overall survival.

ML consists of input and output and is unlike past previously programmed models, in that an ML program learns from the examples and processes massive data. More accuracy can be achieved by training and therefore, more data provides better predictions [32].

Korean Primary Liver Cancer Registry data provided by Korean Liver Cancer Association will be used as input for training an ML model and predicting prognosis of HCC according to preoperative findings and treatment performed. Therefore, for the establishment of a national cohort, the standardization of data and the accuracy of collection must be followed.

By adapting ML to the medical field, increasing amounts of data exceeding that of the capacity of the human brain can be processed in an efficient, time-saving manner. By supplementing records and increasing training sources, the ML model will become an important tool for the selection of appropriate treatment modality for HCC patients in consideration of patient factor, tumor extent and prediction of prognosis. In the future, it will be possible to calculate accurate predictions using a new data set development and differentiated training source for data accumulation. Information on the patient's living environment, economic ability, and social status is also required, and regional and geopolitical locations are recommended to be included as variables.

At the present time, the limitations of developing AI using big data are reliability and missing data. The method of collecting data from various institutions retrospectively has the disadvantage of data not being uniform and the interval between observations inconsistent. It is necessary to simplify and unify the clinical research form of the Korean Society for Liver Cancer. Basic sociological factors should also be included as variables, after which national cohort results can be obtained. It is essential to collect data regularly based on a given template. By using big data collected from multi-centers nationwide, it will be possible to develop a predictive program that provides the basis for treatment response, with factors leading to recurrence after initial treatment. The establishment of a large data cohort of HCC in Korea, which plays a leading role in the epidemiology, diagnosis and treatment of HCC, will greatly advance the development of HCC treatment worldwide. HCC data owned by Pusan ​​National University Hospital will be used to avoid the limitation of data suitability from the multi-center data, which aims to implement a predictive model for the HCC survival rate, survival period, or optimal treatment method based on machine learning..

## Conclusion

With the statistical tools obtained through previous study, an ML program with a deep neural network by deep learning at each layer equipped with the Cox proportional hazard model was analyzed. By developing machine-learning algorithms and deep-learning systems to predict prognosis of HCC patients, it is possible to propose the optimal personalized treatment for each individual according to liver function and HCC status. In order to solve the limitations of multi-center data collected in a standardized form is the priority of national multi-center data management.

## Declarations

### Availability of data and materials

The data were provided by Korean liver Cancer Study Group and the Ministry of Health & Welfare, Korea Central Cancer Registry. The datasets generated and analyzed during the current study are not publicly available due to the authority of this data belonging to Korean Liver Cancer Study Group and Ministry of Health & Welfare’s Korea Central Cancer Registry; data access was granted by the two authorities to the corresponding authors, and can be made available from the corresponding author on reasonable request.
